# A Second New Choroidal Osteoma in the Same Eye: Differences between Them with New Imaging Techniques

**DOI:** 10.1155/2015/684956

**Published:** 2015-03-12

**Authors:** Javier Sambricio, Marifé Fernández-Reyes, Beatriz De-Lucas-Viejo, Álvaro Bengoa-González, Enrique Mencía-Gutiérrez

**Affiliations:** Ophthalmology Department, 12 de Octubre Hospital, Complutense University, 28041 Madrid, Spain

## Abstract

The authors introduce a 42-year-old woman with a choroidal osteoma. After 10 years the patient presented a second choroidal osteoma in the same eye; this osteoma has been growing in the last years. New tests that were unavailable during the first diagnosis were performed such as Fundus Autofluorescence or Enhanced Depth Imaging-Optical Coherence Tomography (EDI-OCT). These tests show characteristics of the tumors and allow us to realize a visual prognosis for the patient.

## 1. Introduction

Choroidal osteoma is a rare benign intraocular tumor that affects the choroid; it is unilateral in 66% of the cases. It manifests predominantly in young women between the second and third decades [1]. It can be asymptomatic, even when it is located subfoveally. However, in the case of choroidal neovascularization or partial decalcification of the tumor [2], a loss of visual acuity and visual field scotoma may be produced. Choroidal osteoma shows growth in 51% of patients after 10 years [2]. Choroidal osteomas show high reflectivity in B-scan ultrasonography. In computerized tomography (CT), a hyperdense plaque is visible in the choroidal plane.

## 2. Case Report

**Figure 1:**
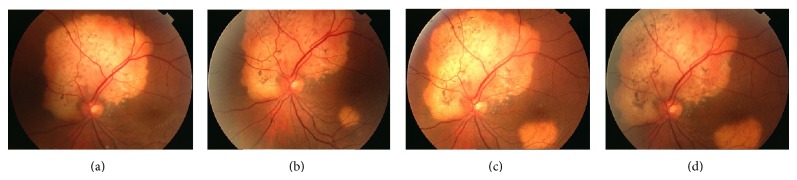
Evolution of the choroidal osteoma during the follow-up: (a) year 2004, (b) year 2011, (c) year 2013, and (d) year 2014.

We present the case of a 42-year-old woman who was diagnosed with choroidal osteoma in the left eye, located in a juxtapapillary position ten years before, with a visual acuity of 20/20. The right eye was unaffected. During follow-up, seven years later, she presented a second choroidal osteoma in the same eye. In this case, the tumor was located in a paramacular position. This second tumor was growing every year, approaching the fovea (Figure 1). Visual acuity remained normal and only a visual field scotoma could be seen in the visual field test. A review of the literature was performed; no similar case was found.

**Figure 2:**
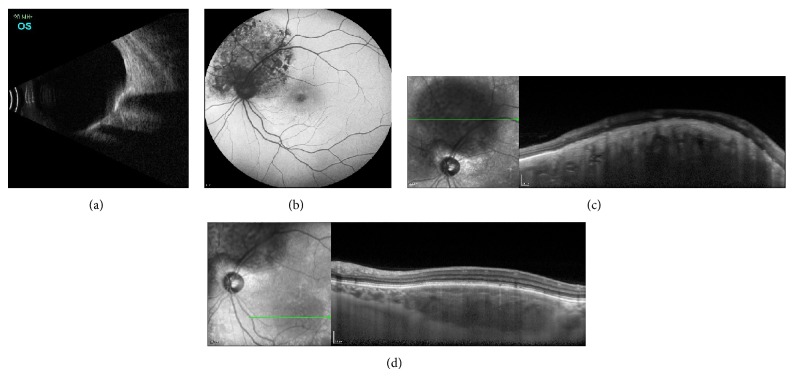
(a) Ultrasound showing a hyperreflective line with acoustic shadowing. (b) Autofluorescence of the left eye. The juxtapapillary tumor presents a hyperautofluorescence and hypoautofluorescence granular pattern. The paramacular tumor presents an isoautofluorescence pattern. (c) EDI-OCT of the juxtapapillary choroidal osteoma showing alterations on the external retina and the sponge-like structure. (d) EDI-OCT of the paramacular tumor shows an intact retina.

Ultrasonography was performed in the affected eye. This technique showed both tumors as a hyperreflective line consistent with bone tissue and acoustic shadowing due to tumor ossification (Figure 2(a)).

## 3. Discussion

Fundus Autofluorescence findings of choroidal osteoma depend on lesion characteristics. There are two different patterns: an isoautofluorescence pattern is observed in the case of total calcification of the underlying tumor. In addition, when the tumor is partially or totally decalcified, a hyperautofluorescence and granular hypoautofluorescence pattern is displayed [3]. In our case, the juxtapapillary tumor presented a hyperautofluorescence and granular hypoautofluorescence pattern due to partial decalcification of this osteoma. In contrast, the paramacular tumor showed an isoautofluorescence pattern, indicating the total calcification of the underlying tumor (Figure 2(b)). This technique leads us to consider that, despite the involvement of the fovea, the visual acuity of the patient will not decrease soon.

In Spectral Domain Optical Coherence Tomography (SD-OCT), there are different retinal statuses depending on the different characteristics of the choroidal osteoma portions: a calcified portion displays an overlying intact inner retina, an intact outer retina, and intact retinal pigment epithelium. On the other hand, a decalcified portion shows an intact inner retina, thinned outer retina, and photoreceptor layers with atrophy of retinal pigment epithelium [4]. In our case, the paramacular choroidal osteoma showed the total calcification pattern (Figure 2(d)). However, the juxtapapillary tumor presented alterations on the external retina and atrophy of the retinal pigment epithelium indicating partial decalcification of the choroidal osteoma (Figure 2(c)).

A new imaging technique, Enhanced Depth Imaging-Optical Coherence Tomography (EDI-OCT), provides a detailed view of the choroid. In choroidal osteomas a sponge-like structure with multilayer configuration is observed [5], as it can be seen in Figure 2. In our case of juxtapapillary osteoma, the external limit of the choroid could not be distinguished due to the large size of the tumor.

In conclusion, choroidal osteoma is a rare benign tumor that may exist both bilaterally and with multiple tumors in the same eye. New imaging techniques provide us with information about the characteristics of the underlying tumor and allow a prognosis of the visual acuity. In our case, the patient developed a second tumor during the follow-up in the same eye. To our knowledge, there is no similar case reported in the literature. Both tumors look similar in retinography; however, with these new imaging techniques, we are able to differentiate which tumor may affect the patient's visual acuity.
